# Biological treatment for bullous pemphigoid

**DOI:** 10.3389/fimmu.2023.1157250

**Published:** 2023-04-27

**Authors:** Meital Oren-Shabtai, Daniel Mimouni, Adi Nosrati, Lihi Atzmony, Baruch Kaplan, Aviv Barzilai, Sharon Baum

**Affiliations:** ^1^ Division of Dermatology, Rabin Medical Center, Petah Tikva, Israel; ^2^ Sackler School of Medicine, Tel Aviv University, Tel Aviv, Israel; ^3^ Adelson School of Medicine, Ariel University, Ariel, Israel; ^4^ Department of Dermatology, Sheba Medical Center, Ramat Gan, Israel

**Keywords:** bullous pemphigoid, autoimmune subepidermal bullous disease, rituximab, dupilumab, omalizumab, corticosteroids, steroid-sparing immunomodulatory agents

## Abstract

**Background:**

Bullous pemphigoid (BP) is the most common autoimmune subepidermal bullous disease. Topical or systemic corticosteroids are often used as the first-line treatment. However, long-term corticosteroid use may lead to significant side effects. Therefore, various adjuvant immunosuppressant therapies are used as steroid-sparing agents, with accumulating reports of biological treatments for severely recalcitrant BP.

**Objective:**

To describe the clinical and immunological features of a series of patients with recalcitrant BP treated with immunobiological therapies. To assess the efficacy and safety of their therapies.

**Methods:**

Patients receiving biological treatment for BP from two centers were assessed. Here, we described the clinical, immunopathological, and immunofluorescence findings of adult patients with BP and analyzed the clinical response and adverse events associated with various biological therapies.

**Results:**

We identified nine eligible patients treated with rituximab (seven), omalizumab (three), or dupilumab (one). The mean age at diagnosis was 60.4 years, the average BP duration before biologic initiation was 1.9 years, and the average previous treatment failure was 2.11 therapies. The mean follow-up period from the first biological treatment to the last visit was 29.3 months. Satisfactory response, defined as clinical improvement, was achieved in 78% (7) of the patients, and total BP clearance was achieved in 55% (5) of the patients at the last follow-up visit. Additional rituximab courses improved the disease outcomes. No adverse events were reported.

**Conclusions:**

Efficient and safe novel therapies can be considered in recalcitrant steroid-dependent BP non-responsive to conventional immunosuppressant therapies.

## 1 Introduction

Bullous pemphigoid (BP) is the most common autoimmune blistering skin disease worldwide, typically affecting the elderly population (over 70 years). BP is chronic, accompanied by significant morbidities, clinically characterized by pruritic tense blisters imposed on normal skin or erythematous urticaria-like plaques, and is caused by circulating autoantibodies targeted at structural dermal-epidermal junction components, such as bullous pemphigoid antigen-1 (BP230) and bullous pemphigoid antigen-2 (collagen XVII, BP180) (1–3). Histologically, subepidermal separation is observed along with an inflammatory infiltrate, often containing eosinophils (4). Linear depositions of IgG +/- C3 are observed along the basement membrane using a direct immunofluorescence assay (5), and autoantibodies are detected in the serum of 60–80% of patients (6). In addition, an indirect fluorescence assay of patient serum with a normal human skin salt split (1 Mol NaCl) demonstrates autoantibodies binding to the epidermal side of the sample (5).

Systemic corticosteroids (SCS) are the mainstream BP therapy; however, their long-term use is associated with significant adverse effects, especially in the elderly (7, 8). Whole-body super-potent topical corticosteroids (TCS) application is effective in patients with BP without the morbidity and mortality associated with systemic administration (9, 10).

Patients who experience a resistant disease and are dependent on long-term steroid treatment require a steroid-sparing immunomodulatory agent, such as tetracycline, intravenous immunoglobulin (IVIG), nicotinamide or dapsone, or immunosuppressive medications (azathioprine, mycophenolate mofetil, cyclosporine, methotrexate, and cyclophosphamide) (11–14). Cumulative data on biological therapies, such as rituximab (RTX)and omalizumab, indicate their clinical benefits in patients with BP (15). In addition, emerging data on BP treatment with dupilumab (DUPI)indicate that it may be an additional immunomodulatory treatment that efficiently controls disease while maintaining a steroid-sparing effect, further expanding the limited existing armamentarium (16, 17).

The exact mechanism by which RTX, omalizumab, and DUPI induce clinical remission in BP has been previously postulated. RTX is a chimeric monoclonal antibody aimed at the CD20 surface protein expressed on B-cell, which produce pathogenic autoantibodies in BP. Immediate lysis of antibody-producing B-cells by effector cells leads to BP remission. During repopulation, nonpathogenic B-cells are generated, which may contribute to long-term clinical remission (18).

Omalizumab is a recombinant humanized anti-immunoglobulin E (IgE) antibody. IgE autoantibodies play an essential role in BP pathogenesis and contribute to tissue damage. IgE autoantibodies directed against the extracellular domain of BP180 bind to FcϵRI on mast cells and eosinophils, causing degranulation and initiating an inflammatory cascade. The binding of specific anti-BP180 IgE to the ectodomain expressed on the basal cells leads to BP180 internalization, followed by adhesion loss and blister formation (19, 20).

DUPI is an IL-4 receptor alpha antagonist, and its efficacy is related to the Th2 inflammatory axis involved in BP pathogenesis. DUPI directly inhibits the activity of IL-4 and IL-13; however, it also downregulates eosinophil chemotaxis and inhibits preactivated B-cell proliferation (16, 21).

In the following case series, we described the clinical and immunopathological features of nine refractory BP patients treated with various biological therapies (RTX, Omalizumab, and DUPI). Additionally, responses to treatment and adverse effects are presented.

## 2 Materials and methods

### 2.1 Patients

Patients from two tertiary medical centers (Rabin and Sheba Medical Centers) in Israel were included in this study under the following criteria (22):

1. Clinical presentation: characteristic pruritic eruption composed of tense bullae and erosions on normal or erythematous skin.
2. Histological findings: lesional skin biopsy stained with hematoxylin-eosin demonstrating subepidermal blister formation and eosinophilic infiltrate.
3. Immunofluorescent assays: one or more of the following:
I. Direct immunofluorescence (DIF) of normal perilesional skin demonstrated linear IgG deposits with or without C3 along the basement membrane zone.
II. Indirect immunofluorescence (IIF) microscopy of patients’ sera with 1 M NaCl-split normal human skin exhibiting IgG autoantibodies on the epidermal aspect of the separated skin.

Patients with other subepidermal blistering diseases were excluded. Patient medical records were reviewed to extract data regarding clinical response and adverse events.

### 2.2 Therapy

Patients were treated non-exclusively with various biologic therapies, including RTX, omalizumab, and DUPI, without any enforced washout period. Patients treated with intravenous (IV) RTX received either the rheumatoid arthritis protocol (2 infusions 1000 mg each, administered 2 weeks apart) or the modified lymphoma protocol (4 infusions of 375 mg/m^2^ 1 week apart), as in pemphigus vulgaris (23). Patients treated with subcutaneous (SC) omalizumab received 300 mg every 4 weeks (24). DUPI administered SC in the regimen approved for atopic dermatitis (AD): 600 mg SC initially, followed by 300 mg SC every other week (25) unless otherwise stated.

Clinical response was assessed using the degree of skin lesions and pruritus, according to the recommendations of an international panel of experts for outcome measures in bullous pemphigoid (26). Disease control was achieved when new lesion formation ceased, established lesions began to heal, or pruritic symptoms began to abate. Complete remission (CR) on minimal therapy is the absence of new or established lesions or pruritus while the patient is receiving minimal therapy for at least 2 months (≤0.1 mg/kg/d of prednisone or minimal adjuvant or maintenance therapy). On the contrary, CR off therapy was without BP therapy for at least 2 months. Partial remission (PR) was defined as a transient new lesion occurrence that healed within 1 week of minimal or off therapy in the same manner. Relapse was defined as the appearance of three or more new lesions in 1 month or at least one large (>10 cm diameter) eczematous lesion or urticarial plaque that did not heal within 1 week in patients who achieved disease control.

## 3 Results

### 3.1 Index case

**Figure 1:**
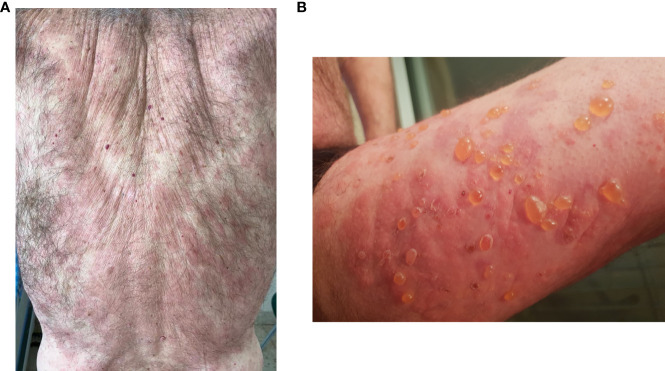
Bullous pemphigoid before rituximab in the index case, the 8^th^ patient. **(A)** Extensive erythematous patches and plaques on the back. **(B)** Tense bullae on erythematous skin on the right thigh.

The 8^th^ patient was a 75-year-old Caucasian male with known dyslipidemia treated with atorvastatin, recently diagnosed with a 6-mm nodular lesion using computed tomography (CT) scan, suspected of renal cell carcinoma. He presented with multiple extremely pruritic erythematous papules and patches (**Figure 1A**), followed by progressive bullae on the trunk and limbs (**Figure 1B**), which developed 4 months prior to his admission to our department. BP was diagnosed based on characteristic clinical features, consistent skin lesion biopsy and histopathological findings (subepidermal blistering with a dermal eosinophilic cell infiltrate), and positive DIF assessment of a perilesional sample (linear IgG and C3 deposition along the basement membrane zone). IIF was negative, yet serological examination *via* a biochip dermatology mosaic 7 (by EUROIMMUN medizinische labordiagnostika, Lübeck, Germany) indicated BP180 positivity on the salt-split normal human skin epidermis. At presentation, his serum IgE level increased to 235 IU/mL (normal range 0–100 IU/mL). The blood eosinophil count was normal, yet it increased later to 3.5 K/μL (normal range 0–0.8 K/μL).

The patient did not respond adequately to twice-daily high-potency TCS whole-body application and oral antihistamines. Thus, he was administered 1 mg/kg prednisone (80 mg/day), with satisfactory clinical response and pruritus alleviation. Nonetheless, the patient repeatedly flared while several SCS tapering attempts were made. After 3 years of intermittently SCS courses, he was diagnosed with iatrogenic osteoporosis, necessitating bisphosphonate treatment and a corticosteroid-sparing agent. The patient responded poorly or suffered from side effects due to prior standard treatment regimens, including doxycycline (100 mg twice daily for 6 months), yielding no efficacy, dapsone (up to 100 mg/day for 6 months), causing significant hemolytic anemia, and non-responsiveness to a lower dose of 50 mg/day and an inefficient azathioprine trial (up to 200 mg/day for 9 months). Therefore, omalizumab was administered in the aforementioned regimen, with no response after 2 months of therapy, and thus ceased.

**Figure 2:**
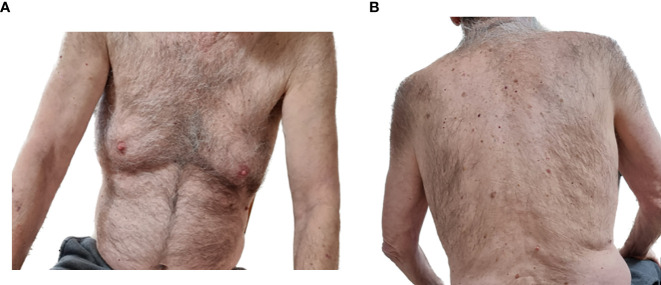
Clinical remission of the bullous pemphigoid three months after the third rituximab infusion in the index case, the 8^th^ patient. No evident skin lesions on the **(A)** front torso or **(B)** back torso.

RTX treatment was administered to achieve disease control. An uro-oncology consultation, based on a repeated CT scan, concluded that there was no contraindication for treatment and that stable solid malignancy requires monitoring only. The patient received his first RTX course at the rheumatological regimen one year after his BP diagnosis while on <0.5 mg/kg/day prednisone. Subsequently, at the 3-month follow-up visit, he reported transient lesions with a minimal itch, and thus concluded as a PR on minimal therapy (prednisone 7.5 mg). Six months after the first course, he experienced a severe relapse, with a disseminated bullous rash and intractable pruritus. A second RTX course was administered, which was soon followed by a good clinical outcome of PR on minimal therapy (prednisone 7.5 mg). After a long remission period of 14 months, another relapse occurred. The third RTX course was administered with complete clearance of skin lesions and no pruritus. Thus, CR was concluded on minimal therapy (prednisone 5 mg). Three months later, the patient maintained this condition (**Figures 2A, B**).

### 3.2 Patient characteristics

Nine patients with confirmed BP diagnosis, according to the clinical and immunopathological features stated previously, were treated with novel biological therapies and included in this case series. The patient demographics and clinical features are outlined in **Table 1**. The mean age of the patients at diagnosis was 60.4 years. None were treated with dipeptidyl peptidase-4 inhibitor (DPP4i).The seventh patient in the series had BP secondary to pembrolizumab treatment due to lung adenocarcinoma, which was crucial and could not be ceased. Other patients received drugs associated with BP. However, the timeline did not indicate it as a possible trigger or exacerbating factor (the third and seventh patients received diuretics, and the fourth received angiotensin-converting-enzyme inhibitors).

**Table 1:** Demographic, clinical, and immunopathological characteristics of patients.

Patient	Sex	Age (years)	Comorbidities	Age at diagnosis (years)	Cutaneous presentation	Mucosal involvement	Diagnosis confirmation
1	M	84	Parkinson, dementia, HTN, BPH	81	Multiple pruritic bullae on the limbs	None	Histology, DIF
2	F	62	Hypothyroidism	57	Multiple pruritic bullae on the trunk and limbs	None	Histology, DIF
3	M	61	Heart failure, DM, HTN, renal failure	52	Multiple pruritic bullae on the face, trunk, and limbs	None	Histology, DIF, IIF
4	F	89	DM, HTN, hypothyroidism, dyslipidemia	79	Multiple pruritic bullae on the scalp, trunk, limbs, and palms	None	Histology, DIF
5	M	22	Noonan syndrome	19	Near erythroderma followed by multiple pruritic bullae on the trunk and limbs		Histology, DIF
6	F	70	Hypothyroidism, depression, and anxiety disorder	62	Multiple pruritic, erythematous papules on the trunk and limbs followed by bullae formation	None	Histology, DIF, IIF
7	F	81	HTN, DM, dyslipidemia, hypothyroidism; Adenocarcinoma of the lung (treated with Pembrolizumab); Chronic urticaria (treated with Omalizumab)	81	Multiple pruritic bullae on the trunk and limbs	Oral and genital	Histology, DIF
8 *Index case*	M	78	Dyslipidemia; Renal cell carcinoma (*requiring follow-up only*)	75	Multiple pruritic, erythematous papules and patches followed by bullae formation on the trunk and limbs	None	Histology, DIF
9	M	42	BCL11B mutation - severe asthma, atopic dermatitis, IgE ~ 8000 IU/mL, developmental delay, recurrent infections. Iatrogenic DM and osteoporosis (steroid dependent).	38	Multiple pruritic bullae on the trunk and limbs	None	Histology, DIF, IIF

DIF, direct immunofluorescence; IIF, Indirect immunofluorescence; HTN, hypertension; BPH, benign prostatic hypertrophy; DM, diabetes mellitus.

Notably, some patients exhibited distinct characteristics. The 5^th^ patient was diagnosed with Noonan syndrome and presented with erythroderma followed by a pruritic disseminated bullous rash diagnosed as BP at the age of 19. However, no association between Noonan syndrome and BP has been reported.

The 9^th^ patient was diagnosed with leaky severe combined immunodeficiecy syndrome due to heterozygous BCL11B mutation. His clinical presentation included marphanoid features, severe asthma (leading to chronic steroid dependence causing iatrogenic diabetes mellitus and osteoporosis), AD, elevated IgE levels (8000 IU/mL), developmental delay, and recurrent skin and respiratory tract infections. He was presented at our department, at 38 years, with a new onset disseminated bullous rash that occurred while he was undergoing SCS treatment, consisting of figurate oval erythematous plaques with tense bullae along the surrounding border. Histology and direct and indirect immunofluorescence analysis were consistent with BP. BCL11B is related to a neurodevelopmental disorder, severe combined immunodeficiency, and AD-like skin inflammation phenotype (27, 28). To date, no known associations with BP have been reported.

### 3.3 Response to therapy

The therapeutic characteristics of patients are presented in **Table 2**. All the patients had refractory diseases which failed to respond to numerous conventional therapies (an average of 2.11 therapies). The average BP duration before biological therapy initiation was 1.9 years.

**Table 2:** Therapeutic characteristics of patients.

Patient	Initial prednisone dose (mg) [Maximal at diagnosis]	Previous systemic BP therapies (other than corticosteroids)	Time from BP diagnosis to biological therapy initiation (years)	Biological therapeutic agent for BP	Biological therapy dosing*	Treatment response at 3-month Follow-up	Time from 1st treatment to relapse and 2nd treatment (months)	2nd treatment response at 3-month Follow-up	Time from 2nd treatment to relapse and 3rd RTX treatment (months)	3rd treatment response/clinical status at the last visit	FU time from diagnosis to last visit (years)	FU time from 1st biological treatment to last visit (months)
1	30	MINO, DOXY, MMF	0	RTX	Rheumatological	CR off therapy				CR off therapy	0.89	2.67
2	40	MINO, MMF	2	RTX	Rheumatological	PR on minimal therapy *(prednisone 5 mg*)	1.76	CR on minimal therapy *(prednisone 2.5 mg*)		CR off therapy	3.72	26.03
3	100	MINO, MMF	2	RTX	Rheumatological	PR on therapy *(prednisone 20 mg*)	12.17	PR on minimal therapy *(prednisone 10 mg*)	22.3	PR on therapy *(prednisone 5 mg and MMF 2g*)	7.18	70.10
4	40	MINO, MTX	1	RTX	Hematological	PR on minimal therapy *(prednisone 5 mg*)	48.70	CR on minimal therapy *(prednisone 5 mg*)		CR off therapy	8.46	89.77
5	50	MINO, DOXY	3	RTX	Rheumatological	NR				NR (*MMF 2g*)	3.47	3.47
6	40	MTX, MMF, MINO	4	RTX > Omalizumab	Rheumatological	NR	7.13 ^**^	NR		CR off therapy; Prurigo nodularis lesions were seen at the last FU visit. Histology and DIF were negative for BP	7.49	42.43
7	30	DOXY	0	Omalizumab	300 mg every 4 weeks	PR on minimal therapy (prednisone 5 mg)				PR on minimal therapy *(prednisone 5mg*)	0.73	4.87
8 *Index case*	80	DOXY, dapsone, azathioprine	1	Omalizumab > RTX	Rheumatological	PR on minimal therapy *(prednisone 7.5 mg*)	6.07	PR on minimal therapy *(prednisone 7.5 mg*)	14.2	CR on minimal therapy *(prednisone 5mg*)	3.16	21.13
9	60	DOXY	4	DUPI	600 mg initially followed by 300 mg weekly	PR on therapy *(prednisone 20 mg, due to steroid-dependent asthma*)	No BP relapse			Treated with 300 mg DUPI weekly for 5 months with relatively good BP control, yet AD and asthma exacerbations led to treatment cessation. Patient received 15 mg of Upadacitinib, leading to AD alleviation. One month later, DUPI 300 mg bimonthly was added due to poor respiratory control. After 3 months, on the last visit, his BP flared.	4.10	3.20

*RTX dosing - rheumatoid arthritis protocol (two 1000 mg infusions each, administered 2 weeks apart) or the modified lymphoma protocol (four infusions of 375 mg/m^2^ 1 week apart).

**Initiation of Omalizumab 300 mg every 4 weeks.

MINO, minocycline; DOXY, doxycycline; CR, complete remission; MMF, mycophenolate mofetil; PR, partial remission; MTX, methotrexate; NR, no response; RTX, rituximab; FU, follow-up; DUPI, dupilumab; AD, atopic dermatitis.

Seven patients received at least one RTX course. Five patients received two RTX courses, and the remaining two received three. Omalizumab was administered to 3 patients, and 1 received DUPI every other week, followed by once weekly administration due to poor response of his asthma and AD. Satisfactory response to biological therapy, implying clinical improvement, was achieved in 78% (seven of nine) of patients at the first 3-month follow-up visit. Three patients maintained their clinical outcome up to the last follow-up visit, while three improved their clinical response, with additional RTX courses administered at that time interval. The last patient with a satisfactory response was the ninth, who maintained a good response on weekly DUPI. However, this regimen was changed due to poor control of his prior steroid-dependent asthma (see below). During the last visit, seven patients achieved a satisfactory response.

Two patients were defined as non-responders at the first 3-month follow-up visit, consisting of the 5^th^ and 6^th^ patients who received a rheumatological regimen of RTX. The 6^th^ patient did not respond to RTX or omalizumab, and yet, was considered to achieve CR off therapy at the last follow-up visit, as prurigo nodularis lesions were observed, with no signs of bullae or erythematous/eczematous plaques. Additionally, histological and DIF analyses were negative for BP. The 8^th^ patient (*index case*) received omalizumab for 2 months without response. Thus, RTX treatment was applied with significant clinical improvement (*the data in* **Table 2** *solely refer to the RTX treatment*).

The mean follow-up period from the first biological treatment to the last recorded visit was 29.3 months. Notably, on evaluating the last visit, 5 patients achieved CR (55%), of which, 4 (44%) achieved CR off therapy, all treated with RTX (4 of 7 patients, 57%). In addition, one patient received a single course, and two received two courses. The sixth patient was treated with omalizumab after a lack of response to the first RTX course (see above, the fifth patient).

Only one patient received RTX as a hematological regimen. Therefore, it was impossible to establish a difference in the effectiveness between both regimens with regard to BP. In 3 patients, additional RTX courses improved the clinical outcome; the 2^nd^ and 4^th^ patients experienced CR on minimal therapy at the 3-month follow-up visit after the first RTX course and were concluded to have CR off therapy by the end of the follow-up after the second course. The 8^th^ patient attained PR on minimal therapy (prednisone 7.5 mg) after the 1^st^ and 2^nd^ RTX courses. After the third course, he achieved CR. Consequently, his daily prednisone dose was lowered to 5 mg.

Two patients required adjuvant therapy with mycophenolate mofetil (MMF) 2 g/day. The 3^rd^ patient was also administered 5 mg prednisone daily, attaining PR on therapy. The fifth patient remained a non-responder. At the last visit, 2 other patients were treated solely with a residual 5 mg of prednisone. The eighth patient *(index case)* treated with omalizumab, followed by three courses of RTX, achieved CR on minimal therapy. Additionally, the seventh patient suffered from chronic urticaria a decade prior to her current BP diagnosis. She was treated with omalizumab for 5 months after the urticarial rash ceased. Soon after the first treatment with pembrolizumab, she developed pruritus followed by a disseminated bullous rash involving the oral and genital mucosal surfaces. SCS provided temporary relief, and doxycycline 100 mg twice daily for 6 months led to minor improvement, yet the pruritus persisted. On the second omalizumab dose, she experienced a significant improvement in pruritus, and on the last visit, she described minor palmoplantar itch, treated with an additional antihistamine, and concluded as PR on minimal therapy. The patient is still receiving lifesaving pembrolizumab therapy, and omalizumab allows her to maintain a good quality of life with minimal pruritus and no blistering.

The ninth patient suffered from the complexity of severe uncontrolled steroid-dependent asthma, recurrent AD, and BP flares, with no treatment thus far to adequately control the three conditions. He was initially treated with 300 mg of DUPI once weekly. Frequent administration was recommended due to the poorly controlled asthma for 5 months, with BP partial remission. Nonetheless, AD and asthma exacerbations led to treatment cessation. Subsequently, the patient received 15 mg of upadacitinib (for 6 months), leading to AD alleviation. One month later, DUPI 300 mg bimonthly was added to achieve respiratory control. Unfortunately, 3 months later, at the last follow-up visit, his BP flared. Notably, the 1^st^ course of DUPI, administered at 300 mg weekly, maintained his severe BP under partial remission. Nonetheless, the asthma was unstable and required a therapeutic change.

Looking at clinical outcomes according to biological treatment, RTX was given to 7 patients (cases 1-6 and 8), among which by the last visit, 3 achieved CR off therapy and one on minimal therapy, two patients required adjuvant therapy with MMF and one did not respond at all, leading to a trial of omalizumab. Among the 3 patients receiving omalizumab (cases 6-8), one maintained CR off therapy at the last visit, and the other PR on minimal therapy, while the third did not respond, thus a RTX course was attempted. DUPI was given to one patient only, concluded to relapse by the last follow-up visit.

The medical records of all the patients were reviewed for adverse events. No adverse events were reported, including infections or allergic/drug reactions. None of the patients discontinued the treatment due to adverse reactions.

## 4 Discussion

This case series describes nine patients diagnosed with recalcitrant BP, necessitating an efficient steroid-sparing agent. Of these, seven patients received RTX treatment. Three were treated with omalizumab and one with DUPI. Satisfactory response, with good control of pruritus and bullae formation, was achieved in 78% (7 of 9) of patients at the first 3-month follow-up visit. At the last follow-up, CR was achieved in 55% of patients (5 of 9), of which, 4 were off therapy, 3 were treated with RTX, and 1 with omalizumab. Additional RTX courses improved the clinical outcomes and enabled further prednisone tapering. Only 1 patient was treated with DUPI 300 mg weekly and achieved good disease control through a 5-month course. The biological treatments were well-tolerated with no reported adverse events.

Recently, Cao et al. evaluated the treatment outcomes of RTX, omalizumab, and DUPI for BP *via* a systematic review of 75 publications, including 211 patients. The mean age of the patients was 68. RTX (122 patients), omalizumab (53 patients), and DUPI (36 patients) had similar clinical benefits in treating BP, with complete remission rates of 70.5%, 67.9%, and 66.7%, respectively. The recurrence rate in the RTX group was higher than that in the omalizumab and DUPI groups. However, the disease duration before treatment initiation was longer. The absence of adverse events in the DUPI group was higher than that in the omalizumab and RTX groups. However, it was similar between the omalizumab and RTX groups (29). This was consistent with a previous systematic review by Kremer et al., which indicated a similar adverse event rate between the two therapeutic agents of 20% and 24%, respectively (15). The main adverse event in RTX treatment was infection (6.6%, 8 of 122), probably related to the mechanism of depleting the B-cell population expressing CD20. Infections were not reported in the other treatment groups; however, the RTX group was significantly larger than the omalizumab or DUPI groups, and the mean age of patients in each group was not stated (29).

Previous studies have indicated RTX as a promising treatment for BP with increased remission rates and steroid-sparing activity (30, 31). Furthermore, Polansky et al. reported that 75% of patients achieved a durable remission after RTX treatment with fewer adverse events and infections after taking RTX than before, suggesting RTX as an alternative treatment for recalcitrant bullous pemphigoid (32).

Omalizumab directly interferes with IgE binding to the cell surface, FcϵRI, leading to BP remission. A subset of patients responds to omalizumab therapy, and biomarkers for predicting good clinical outcomes are currently under investigation (19). The total IgE level correlated with disease severity, yet inconclusively. Furthermore, patients may respond to omalizumab without significantly increasing IgE levels (33). Our index case had mildly elevated IgE levels, though specific anti-BP180 IgE levels were not examined. The patient did not respond to omalizumab. Nonetheless, it might have been too early to conclude, as he was treated for 2 months, with previous reports indicating good outcomes after 4 months of treatment (19) and a mean time to remission of 6.6 months, according to the previously mentioned systematic review (29).

Interestingly, DUPI may improve pruritus by decreasing peripheral itch sensory neuron signaling through its inhibitory effect on IL-4, IL-13, and eosinophils, leading to decreased IL-31 secretion (34).

The seventh patient in this series developed BP secondary to immunotherapy. Cutaneous adverse reaction related with PD-1/PD-L1 inhibitors are common and being increasingly reported. Lopez et al. further characterized immunotherapy-induced BP, stating that pruritus and non-bullous cutaneous findings may be the only symptoms, necessitating awareness and prompt immunofluorescence assays to establish the diagnosis (35).

The most effective treatment of immunotherapy-induced BP has yet to be established. The primary goal is to continue with the immunotherapy treatment and to improve the cutaneous symptoms while enabling a good quality of life. New lesions can appear for months following PD-1/PD-L1 cessation. In the same report by Lopez *at al.*, development of BP required discontinuation of immunotherapy in 76% (16/21) of cases, while the remaining five patients continued the immunotherapy with an additional designated BP treatment. In most cases SCS were the main component of the treatment regimen (35). Muntyanu et al. review the management strategies and recommendations for cutaneous immune-related adverse events of immune checkpoint inhibitors, suggesting starting with whole body application of topical potent corticosteroids twice daily, followed by SCS (0.5-1 mg/kg/day prednisone) intermittently for short periods as possible (36). The optional efficacy reduction of immunotherapy by a concurrent treatment with SCS was postulated, yet not established and further investigation is needed (37).

Other treatment options include dapsone, methotrexate and IVIG. Azathioprine, MMF and cyclosporine should be avoided due to an immunosuppressive effect (36, 38). Antibiotics, such as tetracyclines, should be used with caution. A possible impairment of immunotherapy’s efficiency by an 1-2-months antibiotic course prior to the treatment initiation was postulated (39). At Lopez et al. review five patients were treated with doxycycline with variable cancer outcome, without a causal connection that can be established between the antibiotics and disease progression (35).

Biological therapies, RTX and omalizumab, are also among the treatment recommendations (36, 38). Several refractory immunotherapy-induced BP cases were treated with RTX, experiencing clinical improvement with no established detrimental effect on the malignancy, and even some achieved oncological remission (40–42). Rituximab has been shown to be safe in some malignancies and can act synergistically to other chemotherapeutic agents (43), yet this requires further investigation to a wide range of solid malignancies and combination with immunotherapy specifically. Omalizumab was efficacious as a steroid-sparing agent in a case of a nivolumab-induced BP and was preferred due to evidence of elevated IgE levels. The malignancy outcome is unknown (44).

The limitations of this study include its retrospective nature, small sample size, and the lack of a control group or standardized clinical assessment tool. The mean age of the patients at diagnosis was slightly lower than that reported for BP, and the patients had recalcitrant BP. The group was heterogenic in its medical background, exhibiting unique syndromes and drug associations, which could affect the response to therapies, especially in BCL11B mutation with an atopic predilection, treated with DUPI. Finally, the patients were treated with three biological therapies, which limited the ability to discuss the actual efficacy of each therapeutic agent.

Our study suggests that treatment with biological therapies in severe recalcitrant BP can lead to a satisfactory clinical response, which is significant in persistent cases. Future research should focus on more extensive prospective, randomized controlled trials to evaluate the individual effectiveness and safety of the biological treatment to expand the treatment armamentarium for this disabling disease.

## Data availability statement

The original contributions presented in the study are included in the article/supplementary material. Further inquiries can be directed to the corresponding author.

## Ethics statement

Written informed consent was obtained from the individuals for the publication of any potentially identifiable images or data included in this article.

## Author contributions

DM and SB conceived of the presented idea for this case series. MO-S and AN extracted data from the medical registries and MO-S analyzed the data and wrote the manuscript. All authors contributed to the article and approved the submitted version.
